# Supplementary material for: Identification of photoperiod-induced specific miRNAs in the adrenal glands of Sunite sheep (Ovis aries)
Source: Front Vet Sci. 2022 Jul 22;9:888207. doi: 10.3389/fvets.2022.888207 (PMC9354845; doi:10.3389/fvets.2022.888207)
Supplement: Supplementary file 1 [file Data_Sheet_1.ZIP › Supplementary file/miRDeep2 file.zip!/miRDeep2 file/Know_Structure/SPLP42A3/oar-mir-25.pdf]

miRBase precursor : oar-mir-25
Total read count : 14709
oar-miR-25 read count : 14675
remaining reads : 34

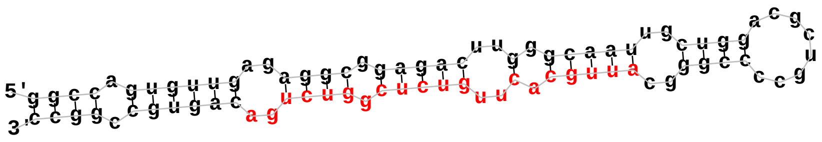

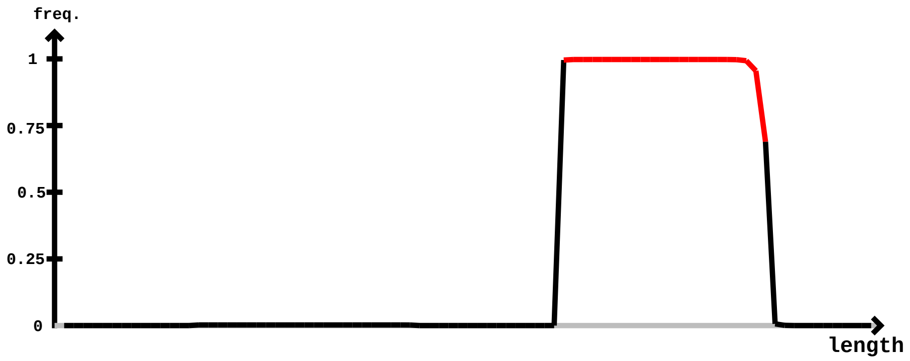

oar-miR-25

```
5'- ggccaguguugagaggcggagacuugggcaauugcuggacgcugccccgggcauugcacuugucucggucugacagugccggcc   -3'    exp
    ((((.((((((..((((.(((((..(.(((((..((((........))))..))))).)..))))).)))).)))))).))))    reads       mm          sample
    .............aggcggagacuugggcaauugc.................................................    3           0           seq
    .............aggcggagacuugggcaauugcu................................................    31          0           seq
    ..................................................gcauugcacuugucucggucug............    1           0           seq
    ...................................................cauugcacuugucucgg................    1           0           seq
    ...................................................cauugcacuugucucggu...............    9           0           seq
    ...................................................cauugcacuugucucgguc..............    58          0           seq
    ...................................................cauugcacuugucucggucu.............    556         0           seq
    ...................................................cauugcacuugucucggucug............    3904        0           seq
    ...................................................cauugcacuugucucggucuga...........    10021       0           seq
    ...................................................cauugcacuugucucggucugac..........    82          0           seq
    ...................................................cauugcacuugucucggucugaca.........    12          0           seq
    ....................................................auugcacuugucucggucu.............    1           0           seq
    ....................................................auugcacuugucucggucug............    8           0           seq
    ....................................................auugcacuugucucggucuga...........    21          0           seq
    ......................................................ugcacuugucucggucuga...........    1           0           seq
```
